# Phylogenetic Analyses Support the Monophyly of the Genus *Lispe* Latreille (Diptera: Muscidae) with Insights into Intrageneric Relationships

**DOI:** 10.3390/insects13111015

**Published:** 2022-11-03

**Authors:** Yunyun Gao, Yingqiang Ge, Liping Yan, Nikita E. Vikhrev, Qike Wang, Nathan J. Butterworth, Dong Zhang

**Affiliations:** 1 School of Ecology and Nature Conservation, Beijing Forestry University, Beijing 100083, China; 2 Zoological Museum of Moscow University, Bolshaya Nikitskaya 6, Moscow 125009, Russia; 3 School of BioSciences, The University of Melbourne, Melbourne, VIC 3010, Australia; 4 School of Biological Sciences, Monash University, Melbourne, VIC 3800, Australia

**Keywords:** *Lispe*, muscid, phylogeny, species group

## Abstract

**Simple Summary:**

While little is known about their phylogenetic relationship, the species *Lispe* are predators inhabiting semi-aquatic environments. Here, we undertook the first comprehensive study to establish the phylogeny of the genus *Lispe*, and to elucidate the intrageneric relationships of the known species group system. The monophyly of the genus *Lispe* is well supported, and the validity of the 11 species group is clarified, while the evolutionary causes remains unclear. This study will provide a better understanding of the phylogenetic relationships and evolutionary history of the genus *Lispe*.

**Abstract:**

*Lispe* Latreille (Diptera: Muscidae) are a widespread group of predatory flies that inhabit semi-aquatic environments. Previous studies on this genus have mainly focused on morphological classification, so molecular data are entirely lacking, and there has been no attempt at a phylogenetic placement of the genus or the resolution of intragenic relationships. To address the phylogenetic placement of *Lispe* and to fill its gap in the Tree of Life Web Project, 58 *Lispe* spp. (covering 11 out of 13 acknowledged *Lispe* species groups) were selected to reconstruct a phylogeny using Maximum likelihood (ML) estimates, Maximum Parsimony (MP) analyses, and Bayesian inference (BI) based on two mitochondrial protein-coding genes (cytochrome c oxidase subunit I (COXI) and cytochrome b gene (CYTB)) and one nuclear gene (a fragment of the carbamoyl phosphate synthetase region of the CAD gene). The phylogenetic analyses indicated that the monophyletic *Lispe* is the sister group of the monophyletic *Limnophora*, together forming the tribe Limnophorini under the subfamily Coenosiinae. Three generic categories are proven obsolete: *Chaetolispa* Malloch, *Lispacoenosia* Snyder, and *Xenolispa* Malloch. Within the genus, the validity of 11 species groups is clarified by both molecular and morphological data. This study provides a sound basis for continuing intergeneric and intrageneric research into this fascinating and widespread genus.

## 1. Introduction

Muscidae (Diptera: Calyptratae) is by far the most species-rich family in the muscoid grade of the Calyptratae, with more than 5000 species from around 180 genera recorded in all biogeographic regions [1,2,3,4]. Remarkably, Muscid flies have conquered both terrestrial and aquatic habitats and have evolved various feeding habits including saprophagy, coprophagy, herbivory, and predation [4,5]. The evolutionary history of the Muscidae has therefore been the focal subject of continuous re-evaluation and reinterpretation at different taxonomic levels (subfamily, tribe, and genus) [3,4,5,6,7,8]. This research has made it possible to establish stable classifications and to begin to understand the evolutionary history of the family as a whole. However, there are still some groups of muscoids that have been largely neglected in this regard; for example, the taxonomic relationships, morphological evolution, and natural history of many aquatic muscoids, such as the genus *Lispe*, remains poorly understood compared to other taxa.

*Lispe* Latreille 1796 is widely distributed in all biogeographic regions with the exception of Antarctica and New Zealand, and includes approximately 200 described species [9,10]. Adult *Lispe* are active predators of small arthropods and also frequently scavenge on arthropod remains [11,12,13,14]. In both larval and adult stages, *Lispe* flies inhabit semi-aquatic environments, ranging from flowing or standing, to fresh or salty, and have thus evolved to tolerate exceptionally variable environmental conditions [15]. Adults are commonly found close to the water bodies, resting or running quickly on mud or sand, in search of mating partners [16,17] and prey. Importantly, *Lispe* play vital ecological roles in the functioning of littoral ecosystems, which makes them useful for monitoring water quality [18]. Yet, despite their ecological, evolutionary, and phylogenetic importance, no comprehensive phylogeny based on modern cladistic analysis has been proposed for *Lispe* as a whole.

The characteristic diagnoses of *Lispe* are as follows: broad frons in both sexes, two upper orbital setae, palpi enlarged from slight to strong, almost spoon-like, setulae present on the anepimeron, katepisternal bristles 1:1 or 1:2 [11,19,20,21]. Within the genus, three sets of morphological traits are particularly useful for specific classification, including thoracic and leg chaetotaxy, leg external modification, and male terminalia [9,11,21,22,23,24,25]. The division of the genus *Lispe* into species groups based on these three sets of characters were offered in several papers [9,11,21,22,24,26,27,28], although all of these studies were only based on morphological approaches.

The first division of *Lispe* into species groups was proposed by Snyder [11], which outlined three Nearctic groups including the *L. tentaculata*-, *L. palposa*-, and *L. uliginosa*-groups [11]. In the work on Palaearctic Muscidae, Hennig [21] agreed with Snyder [11] and expanded Snyder’s species groups with several Palaearctic species, offering three new species groups for the Palaearctic fauna (the *L. caesia*-, *L. longicollis*-, and *L. scalaris*-groups) [21]. Much effort has since been contributed to these six typical *Lispe* groups: *L. tentaculata*-group [21,22,26,28], *L. palposa*-group [9,21], *L. uliginosa*-group [9,11,24], *L. caesia*-group [21,23,24,25], *L. longicollis*-group [21,22,27], and *L. scalaris*-group [21,22]. These six morphological groups are now well acknowledged by taxonomists [9,22,24,26,27,28]. The definitions and developments of these species groups are summarized as follows.

The initial *L. tentaculata*-group is composed of three species (*L. patellata*, *L. sociabilis* and *L. tentaculata*) [21]. More species were eventually added to this group, and then Vikhrev [22] merged the *L*. *nana* complex with the *L. tentaculata*-group as the extended *L. tentaculata*-supergroup [22]. Although Vikhrev [22] had considered the *L. nana*-group as an independent group with a unique knob-like process on the ventral margin of tergite 3 in males and the absence of *pd* seta on the third tibia, it shares many characters with the *L. tentaculata*-group, so Vikhrev [22] also stated: “It is not clear why Hennig [21] had not included *L. nana* Macquart, 1851 in the *L. tentaculata*-group”. The extended *L. tentaculata*-group is characterised by remarkably wide palpi, fore tibia without *p*, mid tibia with 1 *p*, hind tibia with *ad* and weak *pd*, similar abdomen patterns, and sternite 5 with lateral and median processes. All described members of this group seem to inhabit fresh water. Based on two morphological characteristics (first fore tarsomere modification and phallus shape) and molecular data, Ge et al. divided the group into two subgroups (the *L. tentaculata*-subgroup and the *L. orientalis*-subgroup) [28].

The *L. palposa*-group contains more than 20 species in the world [9,21] and most of them live in brackish or salt-water habitats. The main characters of this group are well-defined, such as mid tibia with one or more *ad*, hind tibia without *av* and *pd*, sternite 5 in one piece and either with a shallow median incision or almost concave apically, cerci nearing closely each other along most of their length, but not fused, and the apices rather slender and pointed.

The *L. uliginosa*-group was proposed by Snyder [11] for seven species sharing characters such as the first tibia with *p*, second tibia with 1 *ad* and 1 *pd*, third tibia with 1 *av*, and sternite 5 reduced to a pair of membranous sclerites [9,11,24].

The *L. caesia*-group [21,23,24,25] was first proposed by Hennig [21] and includes five valid Palaearctic taxa, i.e., *L. caesia*, *L. candicans*, *L. halophora*, *L. leucocephala*, and *L. odessae*. Hennig [21] called the *L. caesia*-group one of the most clearly defined, and pointed out the following group characters: a frontal triangle broad with convex margins, femora with ventral rows of short spines, and an abdomen with a characteristic pattern. However, there is an evident discrepancy in Hennig’s approach to the *L. caesia*-group; for example, he included *L. leucocephala*, which has neither a spine on the femora, nor the typical abdominal pattern. Then, some authors extended the volume of the *L*. *caesia*-group with several Palaearctic and Oriental species [23,24,25].

For the *L. Longicollis*-group [21,22,27], members of the old *L. longicollis*-group were divided into two subgroups (subgroup I = *L. longicollis*-subgroup, subgroup II = *L. assimilis*-subgroup) based on morphological differences. According to Vikhrev [27], the subgroup II is further divided into two clades [27]. The first clade consists of *L. glabra* and *L. manicata*, large species with scutum mostly subshining, *dc* 0+2, and *prst* intraalar setae absent. The second clade is *L. assimilis*, *L. nuba*, and *L. pacifica*, all of which are medium sized species with scutum densely dusted, dc 2+4, and prst intraalar setae present.

The *L. scalaris*-group is characterized by *ac* setulae in two rows distinctly separated from scutal setulae, the lower katepisternal setae weak and hairlike [21]. Three species are proposed to form this species-group: *L. persica*, *L. nubilipennis*, and *L. elegantissima*.

In more recent publications, either the volumes of Hennig’s groups have been extended or new groups have been proposed [9,22,23,24,25,27,29,30], such as the *L. bivittata*-group [24], *L. nivalis*-group [22,29], *L. kowarzi* species complex [22], the *L. leucospila*-group [9,22], *L. nicobarensis*-group [9], *L. rigida*-group [9,29], and the *L. pygmaea*-group [30]. It is important to note that Vikhrev [22,30] used a separate term—“complex”—when raising several small groups. We choose “group” and “subgroup” hereinafter to maintain consistent terminology.

Considering the complex radiation of *Lispe* throughout the world and into a variety of semi-aquatic habitats, as well as their remarkable behaviours and ecology, there is much to be gained from a molecular phylogeny that can be used as a foundation for evolutionary questions. To address this, we sample and sequence 58 representative species from the genus *Lispe* (covering 11 out of all 13 proposed species groups), as well as other muscids, aiming to (1) construct the first phylogeny of the genus *Lispe*, and (2) clarify and supplement intrageneric relationships under the current species group system.

## 2. Materials and Methods

### 2.1. Sampling and Molecular Protocols

To investigate the phylogenetic position of *Lispe* in Muscidae and the validity of the currently proposed species group system, a total of 84 calyptrate species were sampled (Supplementary Table S1), covering four species from Fanniidae and Sarcophagidae as outgroups, and 80 muscid species representing all subfamilies. Among these were 58 representative species from the genus *Lispe*, covering 11 out of all 13 proposed *Lispe* species groups. Of all the taxa above, data for 54 *Lispe* species were newly sequenced and documented, although some sequenced data are discarded due to the low DNA quality of specimens (Supplementary Table S1).

For the newly retrieved species, DNA was extracted from part or all of the specimen using different DNA extracting kits following the manufacturer’s instructions and stored at −20 °C until use (Supplementary Table S2). Three genes have been amplified using either published [4,31,32] or self-designed primers (Table 1).

PCR amplification was carried out with 1 μL genomic DNA, 1 μL of each primer (10 μmol/L), 9.5 μL double distilled water, and 12.5 μL 2 × Es Taq MasterMix (Dye) (Beijing Cowin Biosciencee Co., Ltd., Beijing, China). The PCR protocols were just as described before [31]. To ensure the correct PCR amplification, the PCR products were visualized on 1% agarose gels (dyed with Goldeview), then purified and sequenced in both directions by BGI (Beijing, China), as described before [31].

### 2.2. Assembling, Aligning and Nucleotide Substitution Saturation Analysis

Raw sequences were edited and trimmed with BioEdit version 7.0.9.0 [35]. After bidirectional assembling of each fragment using SeqMan (DNAStar, Steve ShearDown, 1998–2001 version, DNASTAR Inc., Madison, WI, USA), assemblies were compared with sequences from GenBank through a blast search to identify the accuracy of data. Sequentially, all genes were aligned individually using MAFFT version 7.3.1 [36] as described before [4]. Each alignment was subsequently examined manually using MEGA version 7.0.26 [37].

We measured whether the sequences were saturated (Index of Substitution Saturation, Iss) and thus useful for the phylogenetic reconstruction based on Xia’s test [38] implemented in DAMBE program version 6.4.81 [39]. In addition, nucleotide substitution saturation of each gene and codon position was evaluated by plotting pair-wise transitions and transversions against divergence for all alignments using the DAMBE following the F84 model. Since third codon positions of protein-coding genes (PCGs) are always the most variable [38], substitution saturation was estimated at the third and combined first and second codon positions of PCGs.

### 2.3. Phylogenetic Analyses

All genes were concatenated together by SequenceMatrix version 1.9.0 [40]. Then, phylogenetic construction was conducted using Maximum Likelihood (ML), Maximum Parsimony (MP), and Bayesian Inference (BI) analyses. For ML analyses, each matrix was subjected to analyses with IQTREE version 1.6.7.1 [41] on its web server [42]. By using the edge-unlinked partition type, the best-fit substitution models for different genes were assigned automatically (Table 2). After tree searching, a standard bootstrap analysis was performed. To estimate the best partitioning scheme and nucleotide substitution models for a BI analysis of each dataset, we used PartitionFinder version 2.1.1 [43] implemented in CIPRES (Cyberinfrastructure for Phylogenetic Research) Science Gateway [44], with “branchlenths” set to unlinked, “models” as mrbayes, “model selection” using aicc, and search for greedy. Then, Bayesian inference was performed with MrBayes v3.2.6 on CIPRES, as described before [31]. The MP analyses were carried out in TNT version 1.5 [45] with the same parameters from a previous study, except for initial addseqs = 13 [4].

### 2.4. Specimen Deposition and Abbreviations

The specimens examined in this study are deposited in the following two museums: MBFU—Museum of Beijing Forestry University, Beijing, China; ZMUM—Zoological Museum of Moscow University, Russia. Abbreviations used for external characters include: *ac* = acrostichal setae; *dc =* dorsocentral setae; *a*, *p*, *d*, *v* = anterior, posterior, dorsal, ventral setae; *prst =* presutural, *post =* postsutural; *tar* = tarsi.

## 3. Results

We present the first comprehensive phylogenetic analysis for the genus *Lispe*, which includes 11 out of 13 currently acknowledged *Lispe* species groups. Four species were used as outgroups (two from Fanniidae and two from Sarcophagidae). Among them, *Sarcophaga forma* and *S. arizonica* were used to root the tree. In total, 315 molecular sequences were submitted to GenBank (see Supplementary Table S1).

### 3.1. Estimation of Substitution Saturation

**Figure 1:**
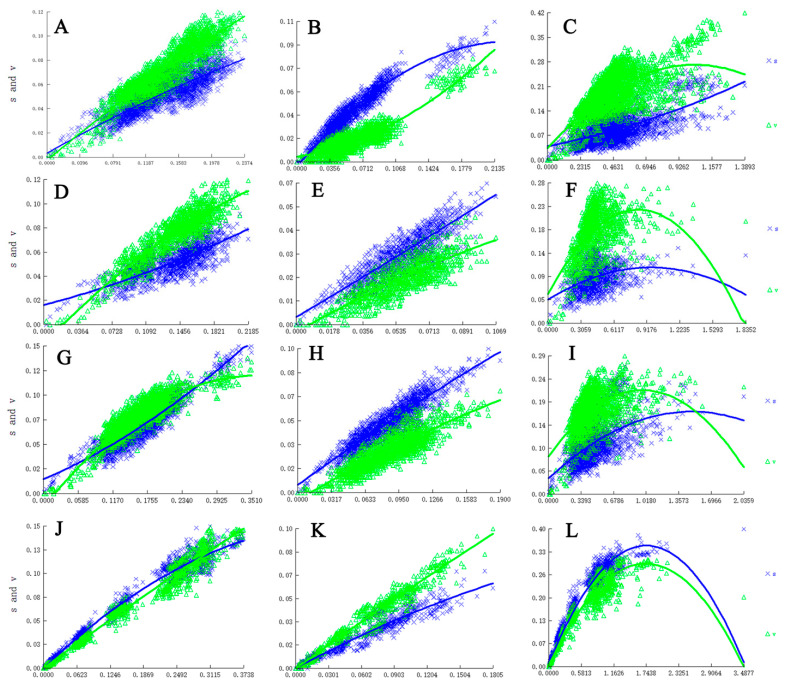
Nucleotide substitution saturation plots of all genes. (**A**). All codon positions of COXIa gene; (**B**). First and second codon positions of COXIa; (**C**). Third codon positions of COXIa; (**D**). All codon positions of COXIb; (**E**). First and second codon positions of the COXIb gene; (**F**). Third codon positions of COXIb; (**G**). All codon positions of CYTB; (**H**). First and second codon positions of CYTB; (**I**). Third codon positions of CYTB; (**J**). All codon positions of CAD; (**K**). First and second codon positions of CAD; (**L**). Third codon positions of CAD.

Our results suggest no or limited saturation of all genes with all codons and all PCGs excluding third codon positions, but with oversaturation at third codon positions of all PCGs (Figure 1). In our analyses, IssSym was selected for assuming a symmetrical topology, and Iss is significantly lower than Iss.c (Table 2). Therefore, all sequences were suitable for phylogenetic analyses.

### 3.2. Phylogenetic Analysis

**Figure 2:**
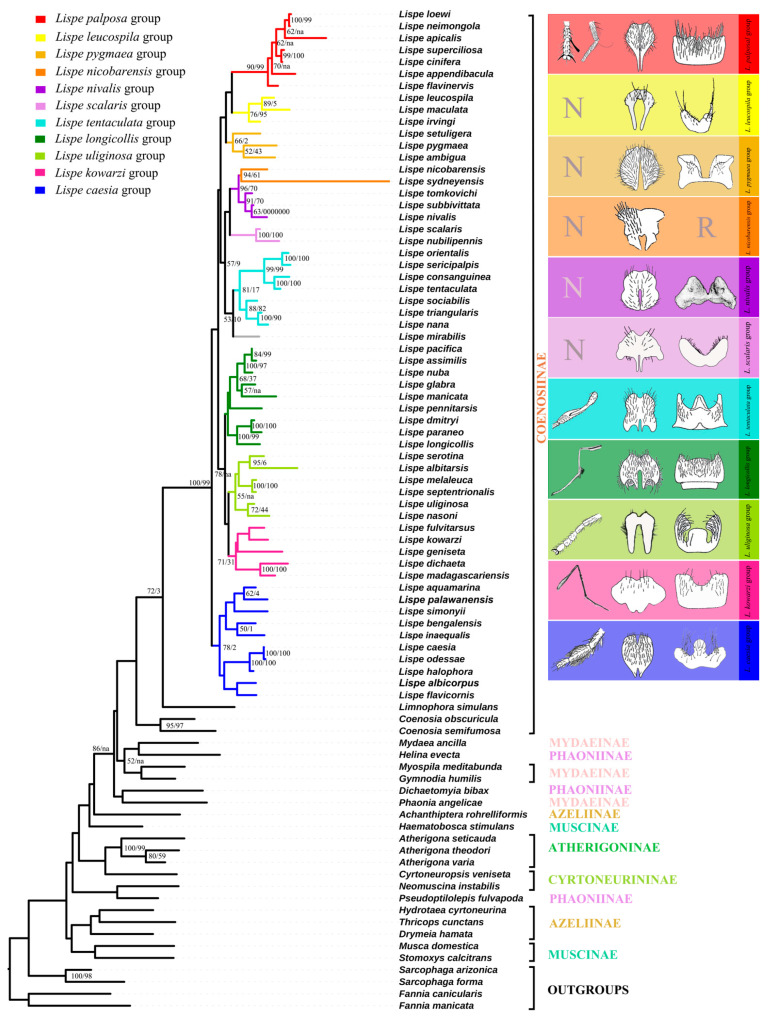
The topologies of maximum likelihood trees based on all selected genes with third codon positions, mapped with morphological and biological characters. N represents the unmodified normal legs. R represents that sternite 5 is reduced. Bootstrap/Jackknife node support values for the ML and MP trees are indicated with on the branches of the tree respectively, bootstrap values less than 50 are not indicated on the tree. Branches indicated with na are not supported on MP trees.

Although topologies resulting from Bayesian inference were overall not well supported (Supplementary Figure S1), we found that both the ML and MP analyses yielded similar results (Figure 2). Coenosiinae form a well-supported monophyletic group (Figure 2) with most subfamilies recovered as para- or polyphyletic. The branch support of Azeliini was weak by bootstrap values (Bootstrap < 50) and denied in MP trees. The Atherigoninae was recovered as monophyletic in consistency with previous research [4]. *Lispe* is inferred as a strongly supported monophyletic group (Bootstrap = 100, Jackknife = 99), and the *Limnophora* was recovered as a sister group to *Lispe* with moderate support (Bootstrap = 72, Jackknife = 3). Thus, with the *Limnophora* as a sister group, *Lispe* are divided into the same 11 clades in accordance with the traditional species group divisions based on taxonomy (Figure 2) with four clades.

Clade 1 contains three species groups: the *L. palposa*-group, *L. leucospila*-group, and *L. pygmaea*-group. The *L. palposa*-group is strongly supported in both the ML and MP analyses (Bootstrap = 90, Jackknife = 99), which is in line with the previous morphological taxonomy (Zhang, 2005). The monophyly of the *L. leucospila*-group [22] is moderately recovered (Bootstrap = 76, Jackknife = 95). The polyphyly of the *L. pygmaea*-group [30] was also proven based on molecular data. Vikhrev [30] divided the old *L. pygmaea*-group into five smaller groups. Among these five groups, the relationships of *L. dichaeta*- and *L. geniseta*-groups are discussed below. Importantly, the *L. pumila*-group is removed from our paper due to its instability and we thus regard it as a rogue taxa. The molecular data indicate a relationship between the two remaining members, the *L. pygmaea*- and *L. ambigua*-groups (Bootstrap = 52, Jackknife = 43). Morphologically, they share the reduced chaetotaxy of a third tibia (1 *ad* seta only). *L. pygmaea* is widespread in the Old World, with *L. ambigua* in the Afrotropical region, while *L. setuligera* is in the Neotropical region. Considering that neither *L. pygmaea* nor *L. setuligera* are distributed in North America, the occurrence of *L. setuligera* in South America is particularly interesting. We tend to integrate the *L. pygmaea*- and *L. ambigua*-groups into the new *L. pygmaea*-group based on the ML tree.

Clade 2 is formed by the *L. nicobarensis*-group (Bootstrap = 96, Jackknife = 70), the *L. nivalis*-group (Bootstrap = 91, Jackknife = 70), the *L. scalaris*-group (Bootstrap = 100, Jackknife = 100), the extended *L. tentaculata*-group (Bootstrap = 81, Jackknife < 50) and the unique species *L. mirabilis*. The topology of these groups in the ML and MP trees is well supported by morphological traits. The molecular data also indicate a close relationship between the *L. nivalis-* and *L. nicobarensis*-groups, which is a new hypothesis that has not been previously suggested. It is, however, not without morphological basis, as the two groups share a sunshining thorax with reduced pollinosity, *dc* reduced to 0+2 or 0+1, reduced leg chaetotaxy, a similar shape of cercal plates (Figure 2) and freshwater habitats. The extended *L. tentaculata*-group consists of the previous *L. tentaculata*-group and the *L. nana*-group (Vikhrev, 2014), which is moderately supported by bootstrap values (Bootstrap = 81, Jackknife < 50) and previously supported by morphological traits [22].

Clade 3 contains three groups, the *L. longicollis*-group, the *L. uliginosa*-group and the *L. kowarzi*-group. For the *L. longicollis*-group, our data showed relatively weak support for its separation into the two former proposed subgroups (the *L. longicollis*-subgroup and the *L. assimilis*-subgroup) (Bootstrap < 50, Jackknife < 50). *L. pennitarsis*, the only genetically examined species from the *L. desjardinsii*-group, is placed in an intermediate position between two subgroups of the *L. longicollis*-group. This result is not unexpected, as the similarity of *L. desjardinsii*- and *L. longicollis*-groups had been proposed by Vikhrev (2014) [22]. For example, both groups have a set of submedian *av*, *ad* and *pd* setae on *t3* and very similar body shapes. Although the validity of the *L. desjardinsii*-group as a whole cannot be verified in this tree, it is suggested that the *L. desjardinsii*-group should be regarded as a subgroup of the *L. longicollis*-group, making up the extended *L. longicollis*-group. The monophyly of the *L. uliginosa*-group is also supported by molecular data (Bootstrap = 55, Jackknife < 50), and the former hypotheses of the *L. uliginosa*- and *L. melaleuca*-subgroups within this group are verified [9]. The relationship of the *L. kowarzi*-group appeared to be enigmatic based on taxonomic research, but the molecular data offer a very interesting hypothesis which combines the old *L. kowarzi-*, *L. dichaeta-* and *L. geniseta*-groups into the new *L. kowarzi*-group (Bootstrap = 71, Jackknife < 50). Such a relationship is also supported by their identical leg chaetotaxy: first tibia with 1 *p*, second tibia with 1 *ad* and 1 *pd*, third tibia with 1 *pd*, 1 *ad* and 1 *av*.

Clade 4 is the *L. caesia*-group [21,23,24,25], which is placed at the base of the *Lispe* genus. The molecular data support the monophyly of the *L. caesia*-group and infer it as the sister group of all other *Lispe* species. However, most species within the *L. caesia*-group are lacking at least one or two of Hennig’s three diagnositic characters. Thus, we offer the following morphological substantiations to redefine the *L. caesia*-group: the presence of at least one of the Hennig’s characters is enough for including species in the *L. caesia*-group, i.e., either *L. leucocephala* with the characteristic frons or *L. bengalensis* with the strong *v* spines on femora.

## 4. Discussion

### 4.1. Generic Relationships within Muscidae

The genus *Limnophora* is proven to be the sister group of *Lispe*, together forming the tribe Limnophorini within the subfamily Coenosiinae in our sampling data. Three genera were proven obsolete: *Chaetolispa* Malloch, *Xenolispa* Malloch, and *Lispacoenosia* Snyder.

The genus *Chaetolispa* Malloch 1922 with type species *C. geniseta* Stein, 1909 and the genus *Xenolispa* Malloch 1922 with type species *X. atrifrontata* Malloch, 1922 (= *Lispe sydneyensis* Schiner, 1868) were both isolated from *Lispe* by Malloch [43,44]. These two genera have not been well recognized by subsequent dipterists [21,45,46]. Our molecular data support the notion that both *Chaetolispa* and *Xenolispa* are groundless and should be merged back into *Lispe*. This result is reinforced by morphology—the diagnostic strong bristle on parafacial close to the lower anterior margin of eyes for *Chaetolispa* is also found in *Lispe* species (*L. dichaeta*, *L. madagascariensis*, and *L. stuckenbergi*). The diagnostic reduced *dc* setae of *Xenolispa* are also shared by some *Lispe* clades (*L. kowarzi*- and *L. nivalis*-groups). Snyder (1949) described *Lispacoenosia fulvitarsus* Snyder, 1949 as a *Lispe*-like Muscidae without the characteristic hairs on the anepimeron [20]. Vikhrev noted that Snyder’s species is very similar to *L. kowarzi*, which also has greatly reduced setulae on the anepimeron occurring as only 1–3 fine setulae, and subsequently transferred it into the genus *Lispe* [22]. Our molecular data confirm that *Lispacoenosia fulvitarsus* belongs to the *L. kowarzi*-group, so the name *Lispacoenosia* should be correctly synonymized to *Lispe*.

### 4.2. Intrageneric Relationships within Lispe

For the *Lispe uliginosa*-group, Vikhrev [9] included one Neotropical species (*L. serotina*) and two Palaearctic species (*L. melaleuca* and *L. septentrionalis*). Both *L. melaleuca* and *L. septentrionalis* were proposed to be sister clades to the other species of the *L. uliginosa*-group [9,24]. Vikhrev [9] also supposed that all American species of the group are descendants of a common ancestor of *L. uliginosa* which colonized America via the Bering land bridge. Molecular data support all of these hypotheses, and the *L. uliginosa*-group can thus be divided into the *L. uliginosa*- and *L. melaleuca*-subgroups.

**Figure 3:**
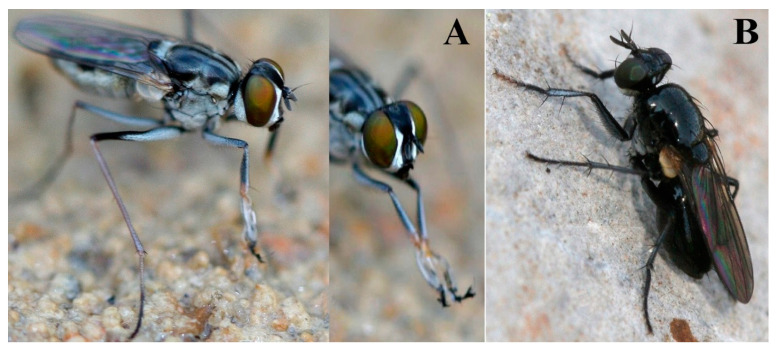
(**A**). *L. mirabilis* Stein, 1918, male, Thailand; (**B**). *L. kowarzi kowarzi* Becker, 1903, female, Turkey (photo: N. Vikhrev).

*L. mirabilis* is an interesting species characterized by strongly reduced chaetotaxy of the scutum and legs (like that in the new *L. nivalis*-group), as well as a modified male *tar* 1-1 (Figure 3) reminiscent of the finger-like protuberance on male *tar* 1-1 in the *L. tentaculata*-group. Although the molecular data supports the sister group relationship of the *L. mirabilis*- and *L. tentaculata*-groups, the true relationship requires further resolution because the former group is only represented by a single species.

### 4.3. Evolution of Morphological Characteristics

The morphological evolution of *Lispe* in terms of three primary traits (external modifications, the shape of cerci, and the shape of sternite) can be traced using the ML tree.

Male terminalia present helpful and reliable information for species identification in numerous dipteran taxa, including *Lispe* [11,21,25,28]. As for flies of small size such as *Lispe*, the dissection of the phallosome into clear divided sub-structures can be difficult. Fortunately, the shape of the cerci and sternite 5 are generally sufficient for group and species level separation (Figure 2). Generally, the gap between the cerci plate increases in size in subsequent descendants of *Lispe* (e.g., *L. uliginosa*-, *L. tentaculata*-, *L. longicollis*-, and *L. pygmaea*-groups).

Various external modifications have evolved throughout *Lispe.* Examples include the swollen hind first tarsomere in the *L. caesia*-group, the merged sternite 4 in the *L. nicobarensis*-group, the flattened fore tarsomeres in the *L. melaleuca* clade of the *L. uliginosa*-group; the finger-like protuberance on the fore tarsomere in the *L. tentaculata* clade of the *L. tentaculata*-group; and the shining black body with limited pollinosity in the *L. nicobarensis*-, and *L. kowarzi*-groups). Interestingly, many of these morphological traits may have evolved in response to sexual selection [17]. This is particularly so for leg morphology and chaetotaxy, because in many *Lispe* species, the leg structures play important roles in courtship behaviour. Males of many species will grasp the wings of females [16] and stridulate their legs on the head of the female, and certain other species will use their legs to position their entire body atop the female (i.e., *L. sydneyensis*) [17].

### 4.4. Origin of Lispe

The origin of the genus *Lispe* is still ambiguous. Vikhrev [22] hypothesized that *Lispe* might have originated from the southern part of the Palearctic region, since the most impressive diversity of *Lispe* is presented in the warmer regions of Asia and Africa. This hypothesis has excluded North America or Australia (both with significant diversity also) as the possible origin of *Lispe* because it is clear that all American or Australian species are descendants of few invaders via the Bering land bridge or dried Torres Strait bridge, respectively [22]. There is, however, a need for more representative samples of Australasian species in future phylogenies, as the region has a rich diversity of at least 39 species [21,45,46,47], including many that inhabit environments ranging from freshwater to salt water. The diversity in the Palearctic region is more impressive than in the Afrotropical region, but it is not so obvious that Africa can be ruled out as the origin of *Lispe*.

Our molecular data support this hypothesis. Among the *Lispe* species-groups proposed by morphological taxonomy, there are several small clades represented only in the Afrotropical region. However, according to our molecular phylogeny, the old *L. desjardinsii*-group is a subgroup of the extended *L. longicollis*-group; the old *L. dichaeta*-group is a subgroup of the new *L. kowarzi*-group; and the old *L. ambigua*-group is a subgroup of the new *L. pygmaea*-group. So, there are no exclusively African clades any longer, and all three new groups include species of both African and Asian origin. At the same time, the large *L. uliginosa* and *L. palposa*-groups are well supported by the molecular data and are represented in the Palearctic region only. In conclusion, the place of origin of *Lispe* is most likely Eurasia, possibly Central Eurasia; specifically, the arid regions between the Caspian lowland and the west part of India.

## 5. Conclusions

This study represents the first comprehensive analysis of the phylogenetic relationships of *Lispe* based on molecular and morphological data and elucidates the validity of 11 species groups of *Lispe*. Although the origin of the genus *Lispe* is still ambiguous, we considered that the origin of *Lispe* is most likely to be Eurasia, possibly in Central Eurasia, and specifically the arid regions between the Caspian lowland and the west part of India. However, it remains unclear as to the causes and consequences of such evolutionary transitions between different habitat types, which itself may require further research into the basic biology and ecology of global *Lispe* species in the future.

## Figures and Tables

**Table 1:** Primers sequences used for PCR amplication and sequencing.

Gene	Prime Sequence	Product Length	Source
COXIa-F	TACAATTTATCGCCTAAACTTCAGCC	~850 bp	Folmer et al. 1994 [32]
COXIa-R	CCCGGTAAAATTAAAATATAAACTTC		
COXIa-F	GGTCAACAAATCATAAAGATATTGG	~750 bp	
COXIa-R	TATGTTTTACCTTGAGGACAAATATC		
COIXb-F	CCACATTTATTTTGATTTTTTGG	~850 bp	
COIXb-R	TCCAATGCACTAATCTGCCATATTA		
COIXb-F	CCWGGATTYGGWATAATYTCT	~700 bp	Zhang et al. 2016 [31]
COIXb-R	CTATGTTCAGCTGGYGGAGTA		
CYTB-F	TAT GTT TTA CCT TGA GGA CAA ATA TC	~800 bp	Kutty et al. 2014 [4]
CYTB-R	AAA TTC TAT CTT ATG TTT TCA AAA C		
CAD-F	GGD GTN ACN GCN TGY TTY GAR CC	~1000 bp	Moulton & Wiegmann 2003 [33]
CAD-R	TTN GGN AGY TGN CCN CCC AT		
CAD-F	TGTGGGTGAGGTTATGGC	~850 bp	Designed using Primer premier 5.0 [34]
CAD-R	CCTCGGAATGTTCCAGTT		

**Table 2:** The loci information, substitution saturation analysis and partitioning scheme for the dataset based on the genes of a basic tree and the respective best models. A: phylogenetic tree for muscoids.

Parameter/Locus	COXIa	COXIb	CYTB	CAD
Alignment length of A	684 bp	765 bp	708 bp	717 bp
Saturation’s test (Iss/Iss.c)	0.389/0.721	0.390/0.729	0.368/0.723	0.382/0.724
Substitution model in ML of A	GTR + F + I + G4	GTR + F + I + G4	GTR + F + I + G4	TVMe + I + G4
Substitution model in BI of A	GTR + I + G	GTR + I + G	GTR + I + G	SYM + I + G

## Data Availability

DNA sequences are open available in National Center for Biotechnology Information (https://www.ncbi.nlm.nih.gov, accessed on 26 September 2022), accession numbers are attached in Table S1.

## Supplementary Materials

The following supporting information can be downloaded at: https://www.mdpi.com/article/10.3390/insects13111015/s1, Figure S1: Phylogenetic tree of Muscidae based on molecular data by Bayesian inference; Table S1: Species used in present analyses with GenBank accession numbers; Table S2: Collection and DNA isolation information of species.
