# Supplementary figures and images for: Phylogenetic Analyses Support the Monophyly of the Genus Lispe Latreille (Diptera: Muscidae) with Insights into Intrageneric Relationships
Source: Insects. 2022 Nov 3;13(11):1015. doi: 10.3390/insects13111015 (PMC9697789; doi:10.3390/insects13111015)

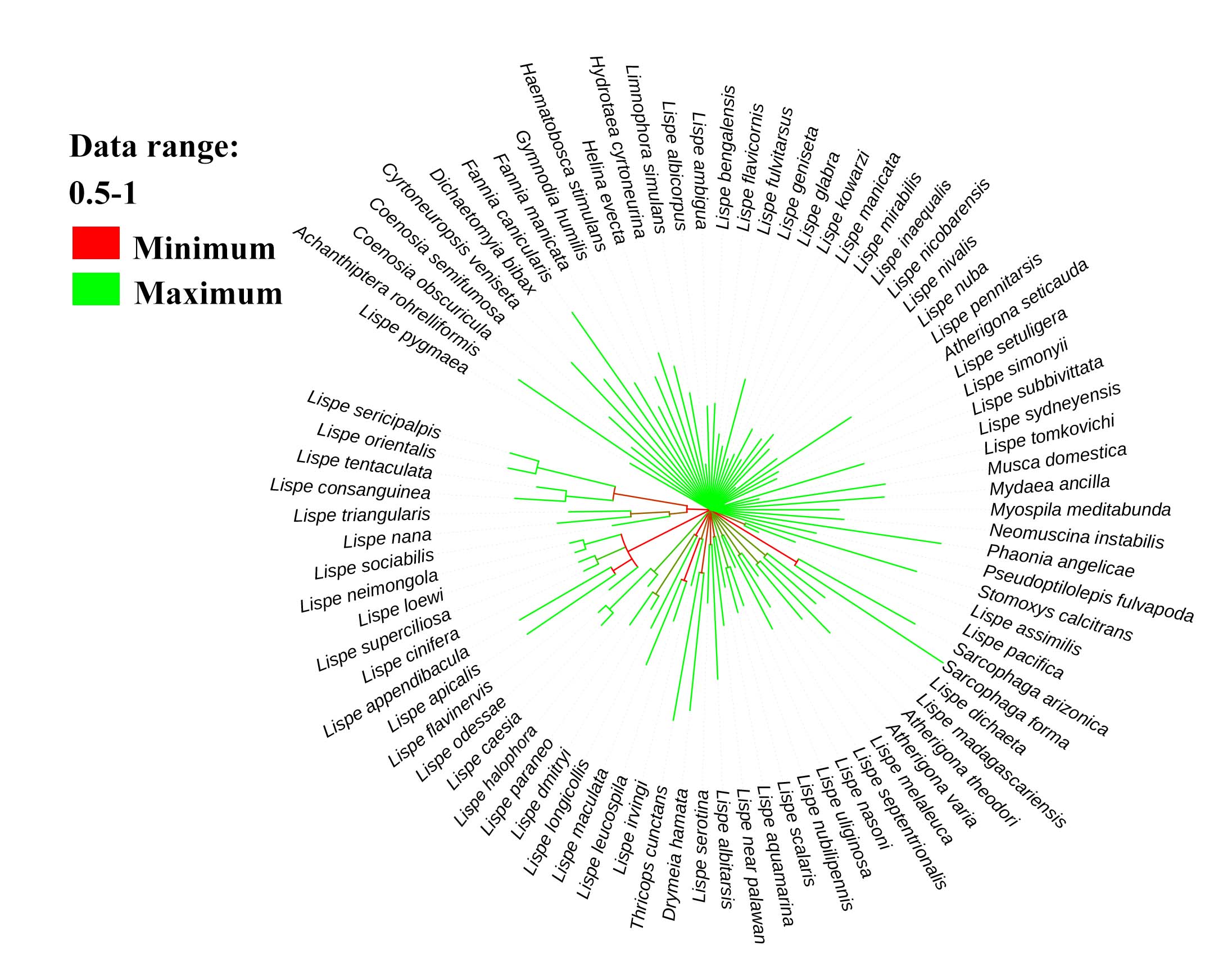

Supplement: Supplementary file 1 [file insects-13-01015-s001.zip › Supplementary FigureS1.jpg]
